# Multi-Class Concrete Defect Classification Using Guided Semantic–Spatial Fusion and Squeeze–Excitation Enhanced DenseNet Model

**DOI:** 10.3390/ma18245665

**Published:** 2025-12-17

**Authors:** Ali Mahmoud Mayya, Nizar Faisal Alkayem

**Affiliations:** 1 College of Automation, Nanjing University of Posts and Telecommunications, Nanjing 210046, China; 2 Computer and Automatic Control Engineering Department, Faculty of Mechanical and Electrical Engineering, Latakia University, Latakia 2230, Syria; ali.mayya@latakia-univ.edu.sy; 3 College of Civil and Transportation Engineering, Hohai University, Nanjing 210098, China

**Keywords:** defect detection, deep learning, DenseNet201, multi-class classification

## Abstract

Concrete materials are vulnerable to various sorts of structural defects. Reliable measurement and quantification of concrete defects are crucial for ensuring safety and effective maintenance. Deep learning is commonly utilized to detect and classify concrete defects efficiently. However, most available studies do not study multi-class defect identification. This study aims to develop a multi-class concrete defect detection framework to enhance concrete classification accuracy while enabling reliable defect localization. To achieve this, a new image-based non-destructive measurement dataset comprising 2029 images of concrete defects, categorized into five categories, has been compiled. For defect identification, the DenseNet201 model is modified by adding a guided semantic–spatial fusion module with a squeeze-and-excitation architecture, which enhances feature representation and introduces attention mechanisms to the model, enabling it to detect and track defect regions. Experiments are conducted on the collected dataset, and various scenarios and comparisons are performed to verify the proposed model. Results reveal the superiority of the proposed architecture with an accuracy enhancement of 5.6% compared to the original DenseNet201. A graphical user interface is also designed to integrate the trained model into a practical measurement instrument, enabling users to interact with the backend model and detect various defects from intact cases.

## 1. Introduction

Concrete structures are the most prevalent type of structure worldwide [1,2]. They often face many inner and outer conditions that can limit their serviceability due to defects [3,4]. Although there are many structural mitigation methods, they fail in severe damage scenarios [5,6]. Moreover, these repair techniques usually require high costs to fix the defects [7]. Hence, early detection of concrete defects is essential [8,9]. In other cases, like earthquake-affected regions, multi-defect detection is necessary to classify the severity of concrete damage [10]. Traditional concrete inspection techniques are based on either subjective visual inspection, which requires expert assessment and leads to expensive and time-consuming methods, or the use of other instruments, including hammer knocking, concrete coring, and ultrasound or laser sensors [11]. Such approaches are either invasive, high-cost, or time-consuming. Current AI technologies that have been effectively utilized address defect identification problems [12]. Additionally, they rely on image-based non-destructive measurements, which require neither invasive instruments nor time-consuming visual inspections, as they are based solely on the camera’s sensors [13]. However, there are still two main issues in the recent defect detection methodologies. The former is the lack of a real and efficient defect dataset, while the latter is the limitations of the utilized deep learning models [14].

When studying the literature, it is found that Hou et al. [15] introduced two lightweight transfer learning-based models for the classification and detection of concrete bridge distress. For this purpose, they utilized two datasets: the Distress Dataset of Asphalt Pavement (DDAP), which contained 2500 pavement distress images, and the Distress Dataset of Concrete Bridge (DDCB), containing 906 concrete bridge images. MobileNet and MobileNet-SSD were trained using those datasets for classification and detection missions. They achieved an accuracy of 97.8% for concrete defect classifications and a mean average precision (mAP) score of 87.16% for the detection scenario. Dinh et al. [13] presented an improved vision-based framework by integrating fracture mechanics (X-FEM) simulations with ML to derive regression models (CRKMDL) of crack shapes. They introduced a crack simulation pipeline and found a strong correlation between the simulation and real concrete cracks. An average classification accuracy improvement of 1.27% was registered using their approach. Although their methodology worked well with curved cracks, they failed to capture other concrete defects, such as spalling or scaling. Moreover, the multi-class crack types were not considered in their study. Fourier enhancement and image augmentation with convolutional neural networks (CNNs) were employed in a study by Sun et al. [16]. They improved the testing accuracy from 87.5% (raw) to 91.6% (median filter) on a dataset of 2828 images collected from public sites and acquired using their own devices. However, they implemented a traditional CNN without any further modification. Moreover, heavy preprocessing operations were sensitive to changes in the image domain, leading to untrustworthy results. Mayya et al. [17] proposed a fusion-based approach combining multiple CNN-based and transfer learning-based models for concrete crack identification. They performed a binary classification operation of a subset of 13,620 bridge deck images selected from the SDNET2018 dataset. As a result, they obtained a test accuracy of 98.62%, a recall of 99.41%, and a precision of 97.64% using the ELM3 model, which was a fusion model of the best CNN and transfer learning-based models. Their study was limited to using bridge deck images and implementing a binary crack classification method. In another study, they developed a fusion-based model that employed YOLOv10 and Vision Transformer (ViT) models for a detection-and-classification fusion mission [18]. YOLOV10 was utilized to detect potential crack regions, while ViT was implemented to classify these regions into their corresponding categories. However, they utilized a detection dataset of 1116 images and a multi-class classification dataset of 12,000 images with only three classes (simple crack, multi-branched crack, and normal). Although experiments showed a good performance of 90.67% for precision, 90.03% for recall, and 90.34% for F1-score, their methodology was limited to the three classes. Transfer learning of many CNN-based models was implemented in a study by Kharthik et al. [19]. In their study, they evaluated 12 pre-trained models (VGG16, VGG19, ResNet50/101/152, DenseNet121, Xception, EfficientNetB0, MobileNet variants, Inception families). Their findings showed that ResNet101 obtained the best accuracy (53.4%) on the SDNET dataset, and EfficientNetB0 registered the best accuracy of 98.8% on the BSD dataset. In comparison, ResNet50 achieved an accuracy of 99.8%, yielding the best performance on the CCIC dataset. However, their study revealed a wide variance across datasets, highlighting dataset bias. A revisiting ViT (ReViT) model was proposed by Zhao [20] for the aim of binary classification of concrete cracks. He trained the ReViT model using his collected dataset, achieving an accuracy of 99.03%. However, this result is limited to his dataset, as it lacks external validation. A multi-class classification method for concrete bridge damage was proposed in a study by Yang et al. [21]. Their proposed method fused image features of a multi-axis vision transformer (MaxVit) model with the label-correlation features of a graph convolutional network (GCN). The utilized dataset consisted of 2098 annotated bridge images with four types of damage (corrosion, spalling, crack, and rebar). They achieved scores of 98.29%, 96.28%, 97.08%, and 96.68%, respectively, in accuracy, precision, recall, and F1-score. The main issue of the study was the limited data size and lack of real-time deployment. In another research, the CrackVisionX model was introduced [22]. The integration of well-known CNN-based architectures, including ResNet-50, DenseNet-121, EfficientNet-B0, and MobileNet-V3-Large, was proposed. Hyperparameter tuning, combined with a data augmentation technique, was also performed to enhance performance. Experiments were conducted on two datasets, SDNET2018 and METU. Binary concrete crack classification was considered in that study, which limited the outcome to detecting the presence of cracks in concrete images. The study registered test accuracies of more than 99% in all scenarios. The limited binary classification framework, which utilized traditional transfer learning models without any modifications, was the most notable limitation of that study. In a study by Lin et al. [23], a Ridgelet deep learning architecture was proposed, combined with a novel evolutionary optimizer (AHEO), median filtering for preprocessing, and a data augmentation operation. They utilized a part of the SDNET2018, a binary classification dataset, consisting of 56,000 images. An accuracy of 99.66%, a precision of 99.194%, and a recall of 99.5% were registered in their experiments. Although their study achieved high accuracy, the binary classification limited their trained models to detecting the occurrence of the crack without knowing its type. Deep belief networks with Ideal Gas Molecular Movement (IGMM) optimization were utilized in a study by Qin et al. [24] for the aim of concrete crack binary classification. The proposed approach achieved an accuracy of 90.189% and an F1-score of 88.093% on the SDNET2018 dataset of concrete images, which has two classes: crack and no-crack.

In terms of research that introduced new datasets, most of the available datasets are limited to the binary classification (crack and no-crack types) [14,15,16,17,19,20,22,23,24]. Several previous studies have addressed the issue of multi-class concrete crack datasets [18,21]. However, in a study by [25], a multi-class dataset was created containing categories such as “General”, which may be confused and not directly related to a specific crack type. There is another comprehensive dataset [26] that addresses the issue of multi-class concrete type, as it includes seven different classes. However, the dataset contains YOLO-based annotations, which are different from the current dataset (classification-based dataset). Other studies considered the utilization of lightweight models for multi-class classification of concrete cracks. Wang et al. [27] gathered two datasets from internet sources, comprising four classes: horizontal, vertical, diagonal, and irregular crack types. They trained the MobileNetV3-C model, achieving an accuracy of 98.73%. Their collected dataset contained 7500 images. Although their study achieved high performance, the collected dataset did not include any challenges, as the geometrical shape of the crack can be easily identified compared to other advanced crack types, such as scaling and spalling. Another modified vision transformer model based on the Cross Swin transformer-skip architecture was proposed by Ye et al. [28]. A binary classification-based dataset consisting of 17,000 crack and non-crack images was utilized. The study demonstrated an accuracy of 96.92%. Although their proposed methodology achieved higher accuracy compared to other methods, they did not consider the multi-class classification problem. Jing et al. [29] presented a hierarchical model for pavement crack detection using point cloud and multi-scale region filtering (F^2^CrackDet-PCD). Their collected dataset, CrackNet-1187, included 502 crack and 95 non-crack point cloud points. As a result, their methodology achieved 95.5% detection accuracy. They did not consider the multi-class classification problem. In another study by Kumar and Ghosh [30], a dual-channel CNN-based model was proposed for the binary classification of concrete cracks using a collected dataset of 3200 images. An accuracy of 92.25% was obtained under various lighting and severity conditions. Many other studies introduced new concrete crack datasets [3,31]. In [31], the authors collected and annotated a dataset of 1132 concrete crack images of both beam and column structures. However, their dataset was limited to binary classification problems. In [3], a multi-class beam crack dataset was gathered. Considering various lighting and environmental variations, researchers collected and annotated 11,123 images of bridge surface defects, including cracks, spalling, seepage, honeycomb surface, exposed rebar, and holes. Although they collected a comprehensive and large dataset, their study was limited to the dataset collection, as they implemented the YOLOV11 model for crack detection without any modifications.

Current studies in the field of concrete crack identification suffer from two main gaps, including the availability of a multi-class concrete defect and crack dataset that includes both crack types (simple or severe) and other concrete issues (spalling and scaling). The second gap is the limitation of CNN-based methodologies to capture all crack and defect variable features (which may be very small in some concrete crack types but large in other defects, such as spalling and scaling).

The main contribution of the current study can be concluded as follows:

- A new multi-class image-based measurement concrete defect dataset for classification tasks is introduced.
- Aiming to significantly improve the concrete defect multi-class identification, a guided semantic–spatial fusion module with squeeze-and-excitation DenseNet201 called (GSSFSEDenseNet201) is designed and implemented.
- New experimental scenarios are conducted using different modifications of the core model to achieve the best-performing model supported by measurement-oriented validation (accuracy, interpretability, and uncertainty analysis).

The next sections will be organized as follows: (i) the developed dataset will be clarified; (ii) the proposed GSSFSEDenseNet201 model will be described; and (iii) the experiments and results will then be introduced and further discussed in the Discussion section. Finally, this research will conclude with a clarification of the current limitations and future perspectives.

## 2. Materials and Methods

During the initial phase of this study, a new dataset comprising five classes of defects was acquired. In the later stages, a novel GSSFSEDenseNet201 model was configured, trained, and evaluated using the collected dataset.

### 2.1. Dataset

**Figure 1:**
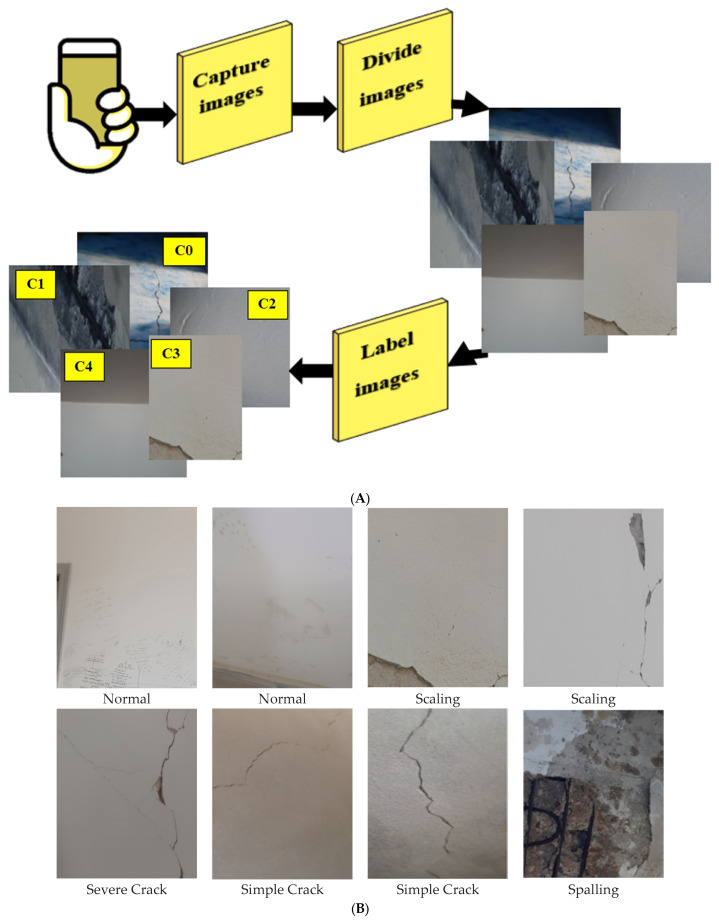
Dataset collection phase: (**A**) the acquisition and labeling process, (**B**) samples of the collected and labeled dataset.

In this study, a new dataset of multi-class concrete crack images has been collected and labeled using different mobile camera sensors with various qualities and resolutions. The collected images were captured under diverse real-world conditions and then cropped and resized. The acquisition process encompassed a diverse range of illumination conditions, including direct sunlight, an overcast sky, shaded regions, and low-light indoor settings. Moreover, other imaging variations, including camera distance, perspective, and surface contamination, were also considered. The presence of crack-like objects (i.e., drawings, writings, cables, etc.) was analyzed. Two specialists in concrete material and its defects labeled the dataset. The utilized annotation protocol defined the criteria used to distinguish simple from severe cracks (like crack width, branching patterns, extent of propagation, and visual depth). This resulted in 2029 images distributed across five different defect types: scaling (403), spalling (415), simple crack (392), severe crack (395), and normal case (424). These numbers indicate that the collected dataset is unbiased with respect to any of the categories it contains. Figure 1A illustrates the dataset collection and labeling phases and some examples of the five classes. The collected dataset presents several challenges, including shadows, illumination variations, crack-like material occurrences, and intra-class variations, as observed in some samples of Figure 1B.

### 2.2. GSSFSEDenseNet201 Architecture

**Figure 2:**
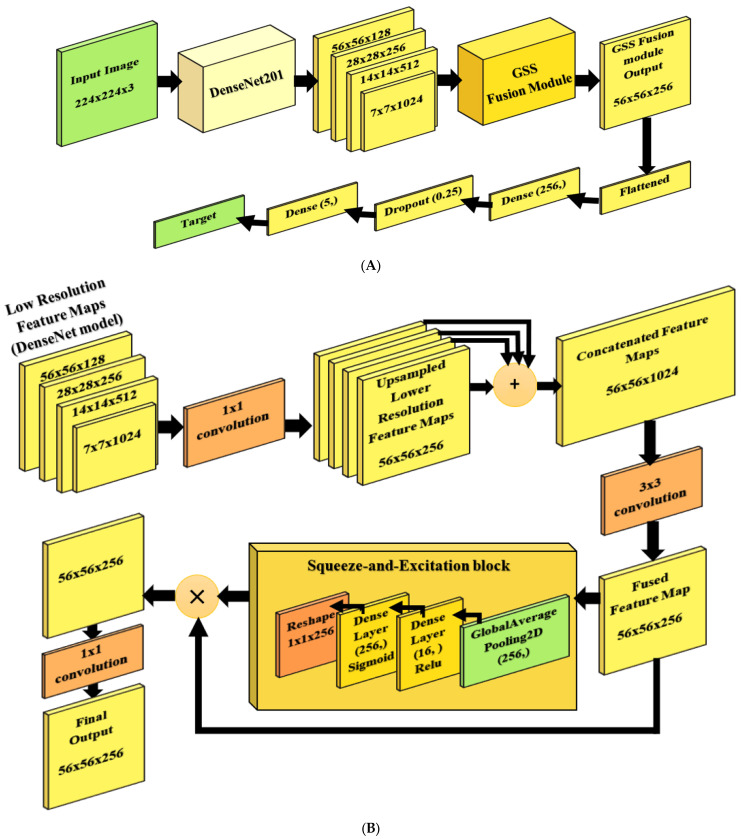
The proposed GSSFSEDenseNet201 framework: (**A**) the overall architecture, (**B**) the proposed GSSFSE module.

The proposed model (Figure 2) is based on the DenseNet201 architecture, supported by two main modules, including the Guided Semantic–Spatial Fusion (GSSFusion) module, which aims to enhance multi-scale feature representation. In contrast, the second is the squeeze-and-excitation (SE) attention block, which forces the model to focus on essential features and drop redundant ones for robust feature representation. The new model is called the GSSFSEDenseNet201 model. The DenseNet201 architecture is chosen as a base model due to its efficient feature reuse, mitigation of the vanishing gradient issue, and regularization. The GSSFSEDenseNet201 model’s novel architectures can be denoted as follows:

- Dual-path feature guidance through the Guided Semantic–Spatial Fusion.
- Cross-attention fusion mechanism and SE-based cross-attention, where channel recalibration is performed after semantic–spatial correlation.
- The GSSFusion module is inserted into DenseNet as a parallel micro-architecture that changes the computational graph of DenseNet.

The main architecture begins with a DenseNet201 model [32] (base model), which accepts input as a 224 × 224 × 3 image, denoted as X. Four feature maps are then extracted from different layers to obtain multi-scale representations. These feature maps Fi are denoted as F1, F2, F3, and F4 and formulated as in (1), (2), (3), and (4).

(1) $$F_{1}=f_{DenseNet,Conv2_{block6_{concat}}\;}(X),$$

(2) $$F_{2}=f_{DenseNet,Conv3_{block12_{concat}}}\left(X\right),$$

(3) $$F_{3}=f_{DenseNet,Conv4_{block48_{concat}}}\left(X\right),$$

(4) $$F_{4}=f_{DenseNet,Conv5_{block32_{concat}}}\left(X\right).$$

These feature maps are then fed into the GSSFusion module to obtain a robust 56 × 56 × 256 fused feature map, which is subsequently flattened into a single-feature vector, denoted as v, and illustrated in (5). A “Dense” layer of 256 neurons with a “Relu” activation function is then utilized as a fully connected layer. After that, a “Dropout” layer with a 0.25 dropout percentage (*p* = 0.25) is used to prevent overfitting and achieve better regularization, as shown in Equation (6). A final “Dense” layer with a “Softmax” activation function is placed as the classification layer, consisting of five neurons (corresponding to the five classes of our problem), as described in (7).

(5) $$v=Flatten\left(GSSFusion\left(F_{i}\right)\right),$$

(6) $$h^{\prime }=dropout\left(Relu\left(W_{d}v+b_{d}\right),\;p=0.25\right),$$

(7) $$\hat{y}=Softmax\left(h^{\prime }W_{f}+b_{f}\right),$$

where Wd and bd are the weights and bias of the “Dense” layer, while Wf and bf are the weights and bias of the final classification layer. Within the GSSFusion module, in Figure 2B, the multi-scale resolution feature maps are subjected to a 1 × 1 convolution phase and then upsampled to a unified higher resolution feature map $F_{i}^{\prime }$ with a shape of 56 × 56 × 256. Equations (8) and (9) clarify the convolution and upsampling steps, respectively.

(8) $${F^{\prime }}_{i}=RELU\left(F_{i}*W_{1x1,i}+b_{1x1,i}\right),$$

(9) $${UF}_{i}={UF}_{s}\left(F_{i}^{\prime }\right),$$

where $W_{1*1,i}$ and $b_{1x1,i}$ are the weights and bias of the ith convolutional layer; “*” refers to the convolution process, where UFi and UFs denote the upsampled maps and the bilinear upsampling operation by a factor of s. After that, the upsampled feature maps are concatenated, CATmaps=[UF1;UF2,UF3,UF4], to form a unified 56 × 56 × 1024 feature map. The unified feature map is convolved using a 3 × 3 convolution step, and the resulting fused feature map Fmap, as shown in Equation (10), which is of size 56 × 56 × 256, is fed into the SE block.

(10) $$F_{map}=RELU\left({CAT}_{maps}*W_{3x3}+b_{3x3}\right),$$

The output and input of the SE block (SEout) are then multiplied to configure a 56 × 56 × 256 feature vector (CFM), which is convolved using a 1 × 1 convolution layer, to produce the final feature map (FFM) of size 56 × 56 × 256 as illustrated in Equation (12).

(11) $$CFM=F_{map}\times {SE}_{out},$$

(12) $$FFM=RELU\left(CFM*\;W_{1x1,fse}+b_{1x1,fse}\right),$$

where $W_{1x1,fse}$ and $b_{1x1,fse}$ are the weights and biases of the final layer of the SE block. The SE block comprises a global average pooling (GAP) layer, a dense layer of 16 neurons with a “Relu” activation function, and another dense layer of 256 neurons with a “Sigmoid” activation function, followed by a reshaping layer to obtain the output size (1 × 1 × 256). The GAP layer helps to compress the input feature map using a spatial averaging operation of each channel in the fused map (Fmap), as illustrated in Equation (13).

(13) $$GA=\frac{1}{H\times W}\;\sum_{h=1}^{H}\sum_{w=1}^{W}F_{map},\;$$

The excitation part (SEout, as denoted in Equation (14)), which consists of two dense layers with non-linear activation functions, is responsible for learning channel-specific weights. The multiplication operation, applied later, is intended to reweight the original feature map to capture the new learned importance (attention).

(14) $${SE}_{out}=\sigma (RELU\left(GA\times W_{1}+b_{1}\right)W_{2}+b_{2}),\;$$

where W1, b1 and W2, b2 are the weights and biases of the first and second “Dense” layers, while σ refers to the sigmoid activation function of the second “Dense” Layer.

### 2.3. Performance Evaluation Measurements

Assessment of DL models is essential to judge the trained model’s robustness, including the computation of accuracy, precision, recall, F1-score, area under the curve (AUC), and confusion matrix (CM) [33,34,35]. AUC is the area under the Receiver Operating Characteristic curve (ROC), in which the true and false positive rate relationship is represented. With high AUC across all thresholds, the model effectively differentiates between positive and negative samples. The CM presents detailed calculations of the precision and recall metrics of all classes. In our mission, the CM helps define classes with the fewest misclassifications, as well as analyze error patterns by identifying pairs of classes that frequently share misclassified samples. Table 1 includes the standard mathematical formats and definitions of each metric.

## 3. Results

### 3.1. Model Training Options

Experiments of this research are conducted using the COLAB environment with a Tesla T4 GPU and Python programming language. Table 2 concludes the training parameters and their corresponding values. The training process utilizes the “Adam” optimizer with a learning rate of 1 × 10^−3^ and categorical cross-entropy as the loss function, as we are dealing with multi-class classification problems. The training lasts for 50 epochs, with an early stopping condition in which the validation accuracy is monitored; the training is stopped if the validation accuracy does not improve for 15 consecutive epochs. The input images are of size 224 × 224 × 3, and the chosen batch size is 128 for optimal utilization of the GPU hardware.

### 3.2. Training and Validation Results of the Proposed

**Figure 3:**
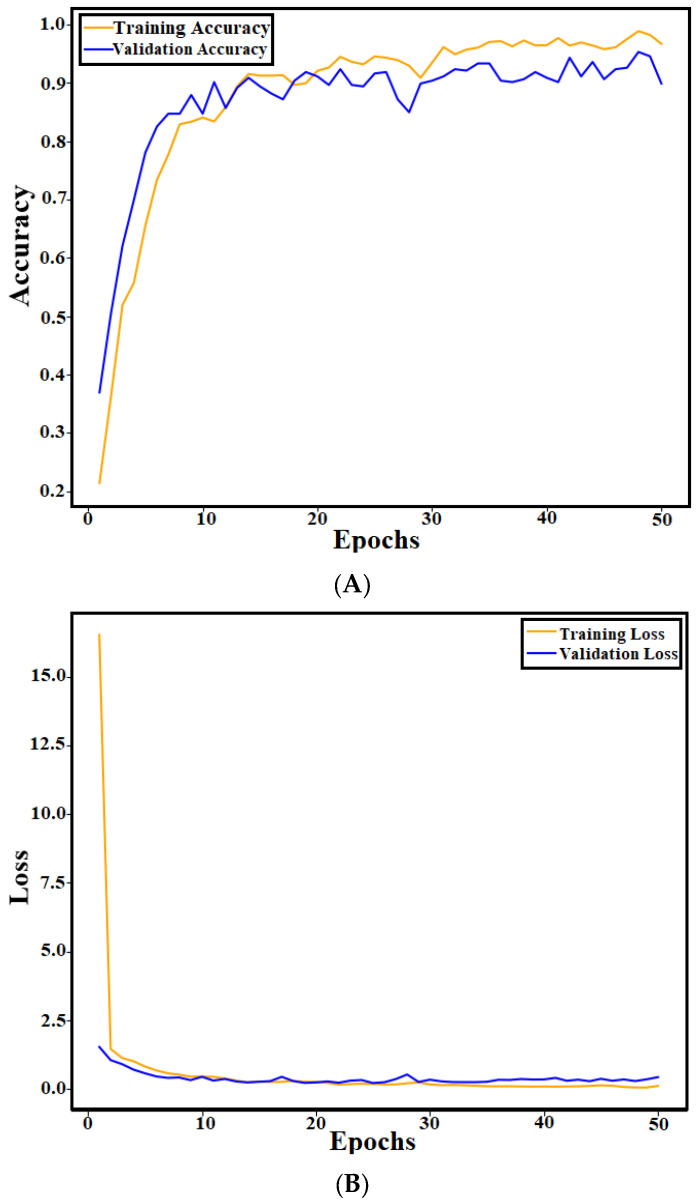
Training and validation curves of the GSSFSEDenseNet201 model: (**A**) accuracy curves; (**B**) loss curves.

In the training phase, the proposed GSSFSEDenseNet201 model is trained using the following scenario: First, the dataset is split into a training set comprising 80% of the data and a validation (test) set containing 20%. The data augmentation operations are also performed on the training set to enhance the training process by increasing the dataset size and allowing the model to learn variations within the training set, which leads to improved robustness and generality. The proposed data augmentation operations include rotation with a range of 10 degrees, zooming with a range of 0.15, and random horizontal and vertical flips. Moreover, the training images are shuffled for each epoch for a better learning process. The training accuracy, training loss, validation accuracy, and validation loss curves are derived from the training process and shown in Figure 3. The curves indicate no overfitting and good convergence performance.

Table 3 includes the detailed performance calculations of the trained GSSFSEDenseNet201 model. The “Normal” class achieves the best performance across all metrics, while the “Scaling” category exhibits the worst precision, at 0.9286. On the other hand, the “Severe crack” category scores the worst recall value of 0.8750. The overall validation accuracy of the trained model is 0.9532, while the overall loss is 0.2915. The registered average precision, recall, and F1-score are 0.9517, 0.9489, and 0.9501, respectively. The high precision and recall values indicate the model’s ability to address both false positive and false negative errors, preserving a balanced trade-off between the two metrics.

**Figure 4:**
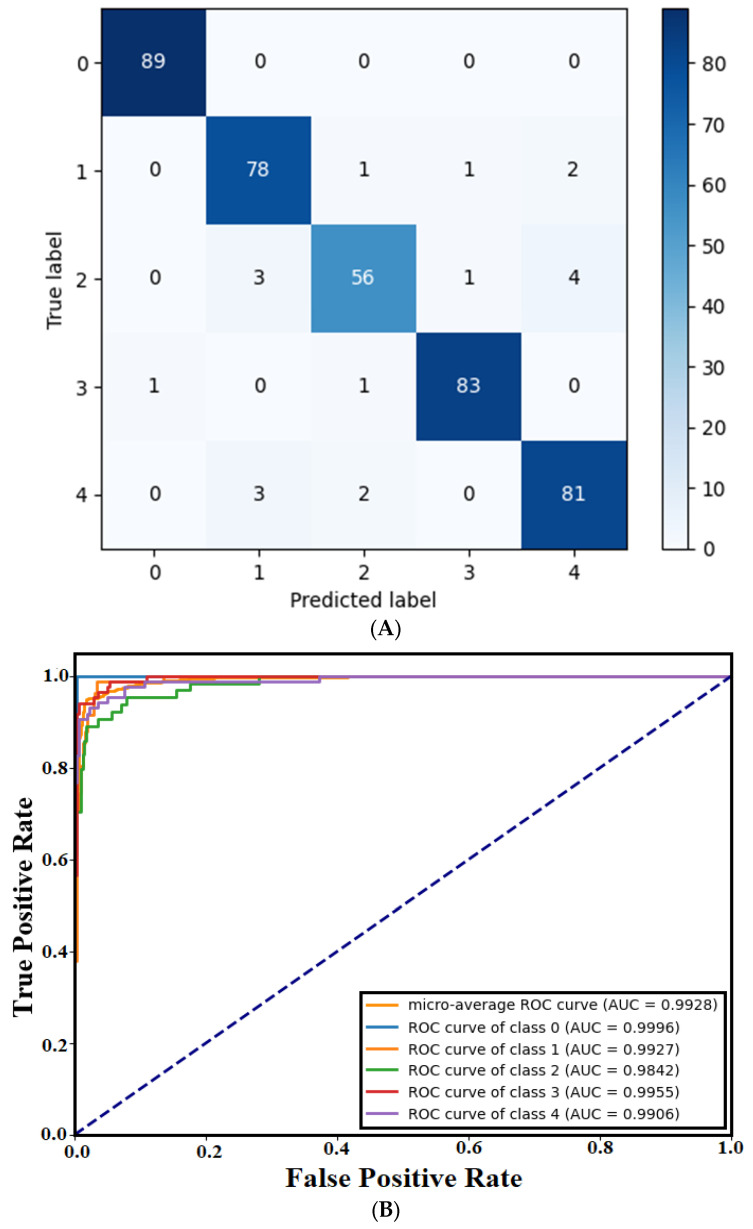
Trained GSSFSEDenseNet201 model’s graphical results: (**A**) CM, (**B**) ROC Plot.

The confusion matrix (CM) of the trained GSSFSEDenseNet201 model, which describes the class-wise prediction performance, is shown in Figure 4. CM indicates that the “Normal” class (index 0) has one prediction error (a sample of a “Simple crack” which is misclassified as “Normal”). Three samples of the “Severe crack (index 2)” category are incorrectly accepted as “Scaling (index 1)”, and three other samples of “Spalling (index 4)” are also misclassified as “Scaling”. In comparison, four samples of the “Scaling” category are misclassified as belonging to the “Severe crack (index 2)”, “Spalling”, and “Simple crack (index 3)” categories, respectively. In terms of “Severe crack”, eight false predictions are registered as “Scaling”, “Simple crack”, and “Spalling”, putting this category in the first worst place in terms of recall. However, only four wrong predictions of categories “Scaling”, “Simple crack”, and “Spalling” are noticed. The “Simple crack” class has two misclassifications and is sometimes confused with visually similar categories. The “Spalling” class includes five misclassified instances distributed across “Scaling” and “Severe crack” defect types. The ROC plot and AUC score reveal that the best registered AUC score corresponds to the “Normal” case, which is equivalent to the numerical results. An average AUC score of 0.9928 is also registered.

**Figure 5:**
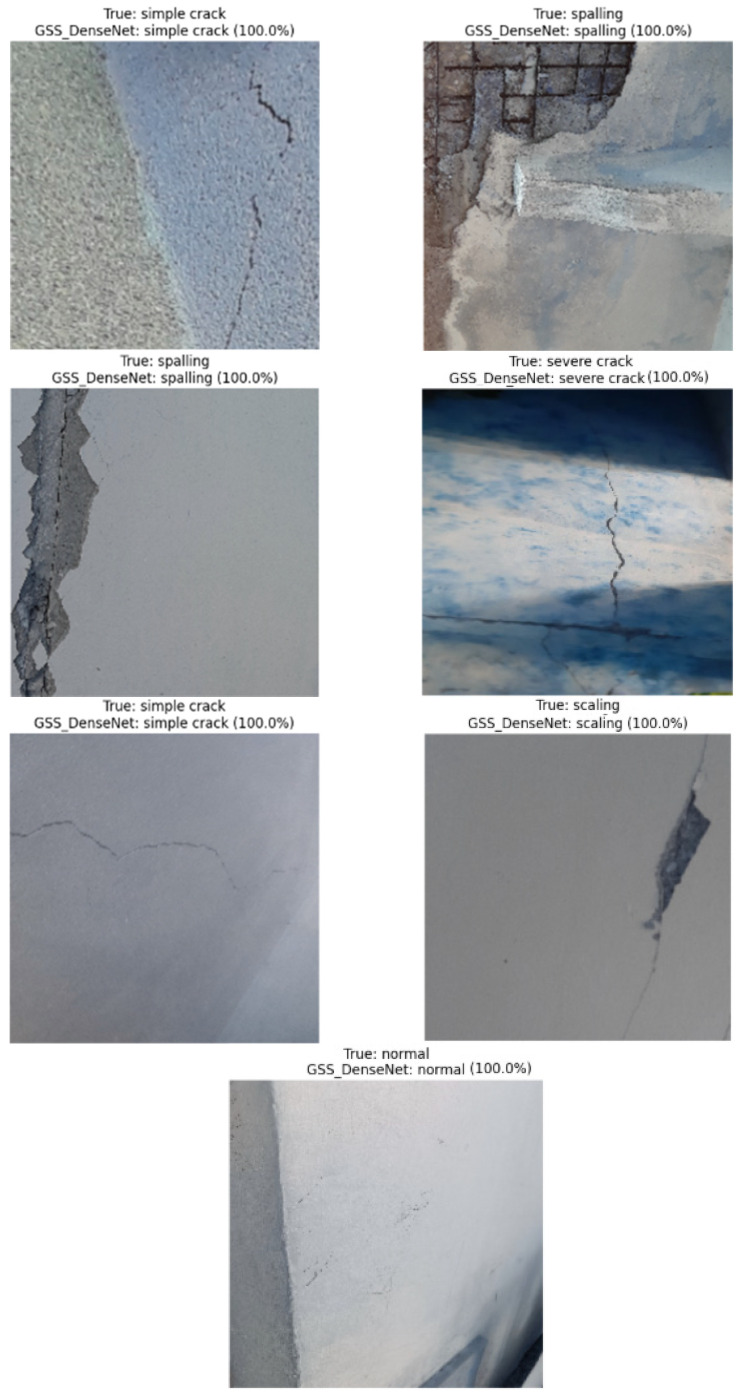
Visual tests of the proposed GSSFSEDenseNet201 model using some test samples.

Some graphical results are shown in Figure 5, which prove the efficiency and robustness of the proposed GSSFSEDenseNet201 model. The model can accurately identify the type of crack, even in the presence of shadows, crack-like effects, intra-class variations, or texture variations.

## 4. Discussion

A comparison of the proposed GSSFSEDenseNet201 model with the original DenseNet201 model is shown in Table 4. The proposed GSSFSEDenseNet201 model outperforms the original DenseNet201 model by 5.66%. The macro average precision, recall, and F1-score of the proposed model exceed the original model by 5.91%, 5.51%, and 5.76%, respectively. The most affected categories by such enhancements are the “Scaling” and “Severe crack” types. The “Scaling” category’s recall is enhanced by 10.97%, while the precision score of the “Severe crack” is improved by 15.87% compared to the original DenseNet201 model’s scores.

**Figure 6:**
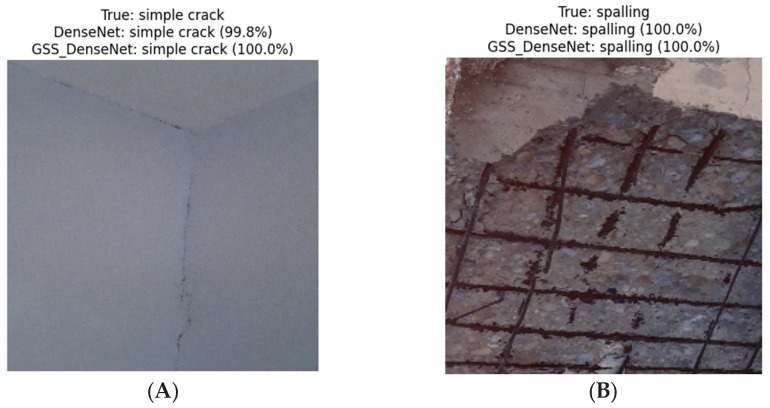

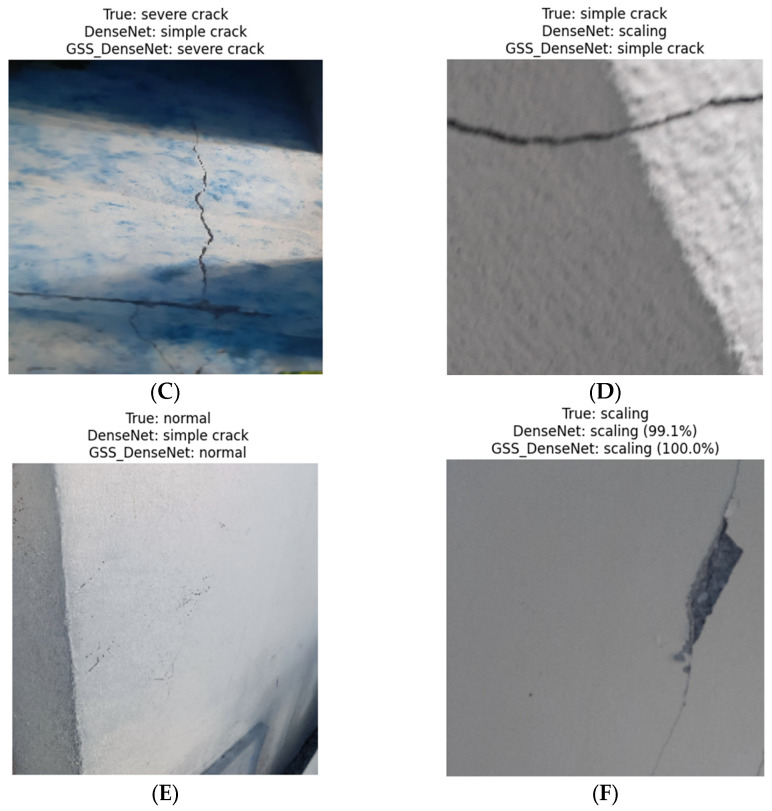
Comparison between the proposed and the original DenseNet201 model in some prediction examples of the test set. (**A**) Sample 1; (**B**) Sample 2; (**C**) Sample 3; (**D**) Sample 4; (**E**) Sample 5; (**F**) Sample 6.

Figure 6 shows a visual comparison between the proposed GSSFSEDenseNet201 model and the original DenseNet201 model. The comparison indicates that illumination variations (Figure 6C) limit the DenseNet201’s ability to recognize the “Severe crack” type. In contrast, Figure 6D shows that the DenseNet201 model incorrectly classified the “Simple crack” type as “Scaling”. Again, in the “Normal” case, illumination variation adds a crack-like texture; the DenseNet201 model failed to classify the sample compared to the proposed GSSFSEDenseNet201 model, as shown in Figure 6E.

### 4.1. Ablation Study: Modifying the DenseNet201 Architecture

**Figure 7:**
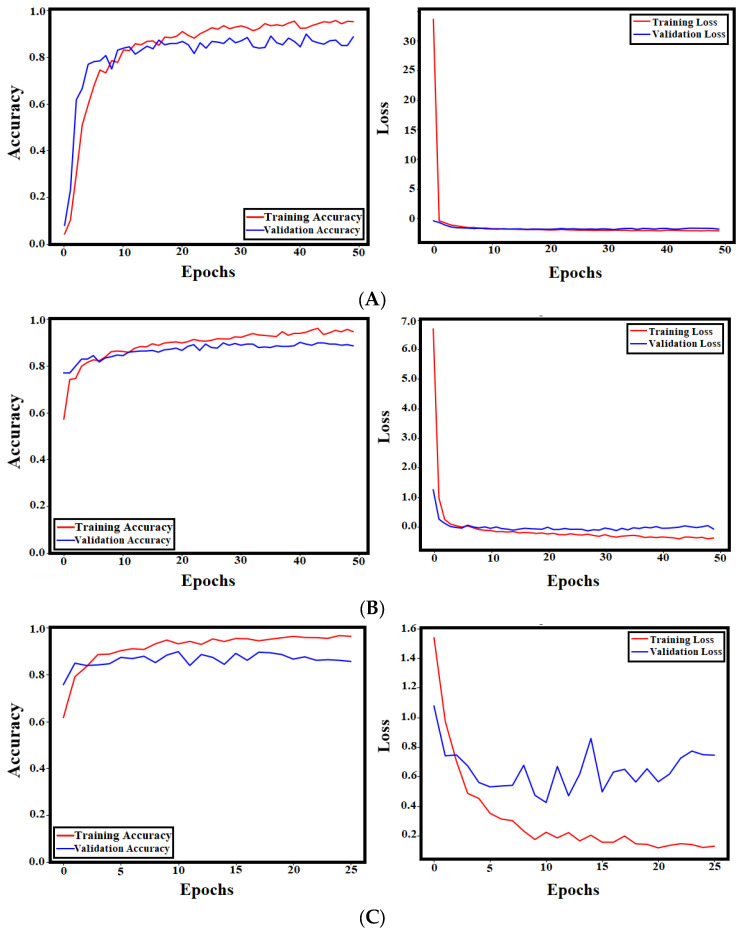
The training and validation accuracy and loss curves for all models: (**A**) GSSFDenseNet201; (**B**) DenseNet201 with dual attention; (**C**) DenseNet201.

To prove the efficiency of the proposed methodology, the SE module is removed and the model is retrained. Moreover, another version of the DenseNet201 model has been designed and trained to compare the proposed architectures. The modification of DenseNet201 involves incorporating dual attention techniques (spatial attention and channel attention) into the original model. Table 5 presents the detailed results of the proposed model after eliminating the SE block and the dual-attention-based DenseNet201 model. Table 5 demonstrates that the DenseNet201 with the dual attention model enhances the accuracy of the DenseNet model by only 0.24%, which is significantly less than the proposed GSSFSEDenseNet201 model’s enhancement of 5.6%. Figure 7 presents the training and validation curves for all models, which supports the same conclusion.

The learning rate tuning is also considered in the ablation study to select the best option precisely. Table 6 presents three trials using different learning rates for training the GSSFSEDenseNet201 model. Results indicate that 0.001 is the optimal learning rate for the proposed model.

### 4.2. Misclassified Samples

**Figure 8:**
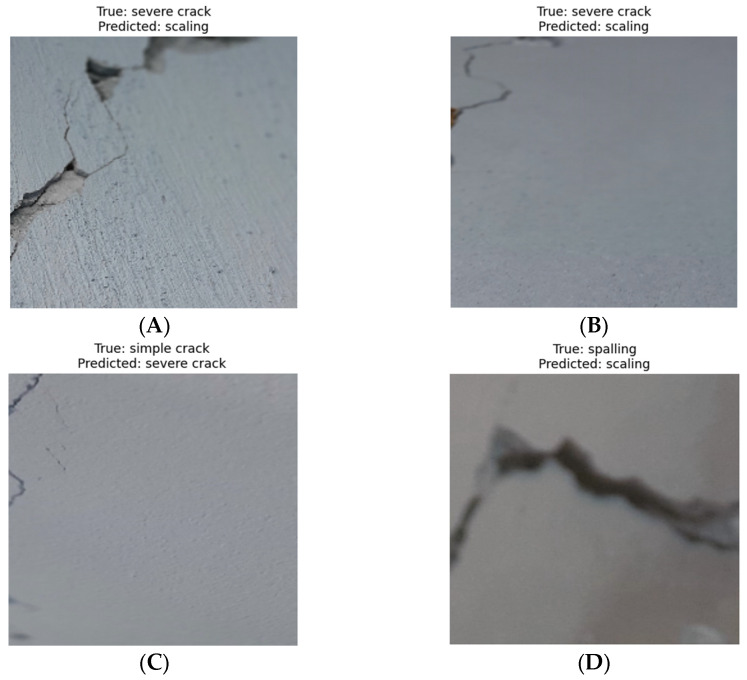
Examples of misclassified samples. (**A**) Sample 1; (**B**) Sample 2; (**C**) Sample 3; (**D**) Sample 4.

To explore the leading causes of misclassification errors in the proposed GSSFSEDenseNet201 model, Figure 8 presents examples of these false positives and negatives. Figure 8A,B includes two samples of the “Severe crack” type, while the model predicts them as “Scaling”. In the first case, the “Scaling” effect is present in three parts of the image, and the crack is also visible; therefore, the annotator labeled it as “Severe crack”. In Figure 8C, the occurrence of many simple cracks biased the decision of the proposed model to be “Severe crack”. In the last sample, the model incorrectly predicts the “Spalling” sample as “Scaling” because the blurring removed the main texture of spalling. As we summarized in the results of the confusion matrix’s numbers, the most frequent error pattern occurs between “Scaling” and “Severe crack” (three errors with a percentage of 4.69%) and between “Severe Crack” and “Spalling” (four errors with a percentage of 6.25%). The spalling-to-scaling error percentage is the next one, with a score of 3.49%, while the “Normal” category has a 0% misclassification percentage. The leading cause of such misclassifications is that some image samples may contain elements of both categories (“Scaling” and “Severe crack”). Still, the sample must have only one label, so the annotation process assigns the class that is closest to the sample. Another cause of the “Scaling and Spalling” error is the similarity between these two categories, which can even confuse experts themselves. Possible improvements include creating bounding box annotations that enable the classification of multiple categories within the same image. However, this requires distinct annotations and other object detection models, such as the YOLO model. Other possible improvements can be achieved by applying preprocessing operations to the images (i.e., noise reduction and contrast enhancement).

### 4.3. Real-World Application

**Figure 9:**
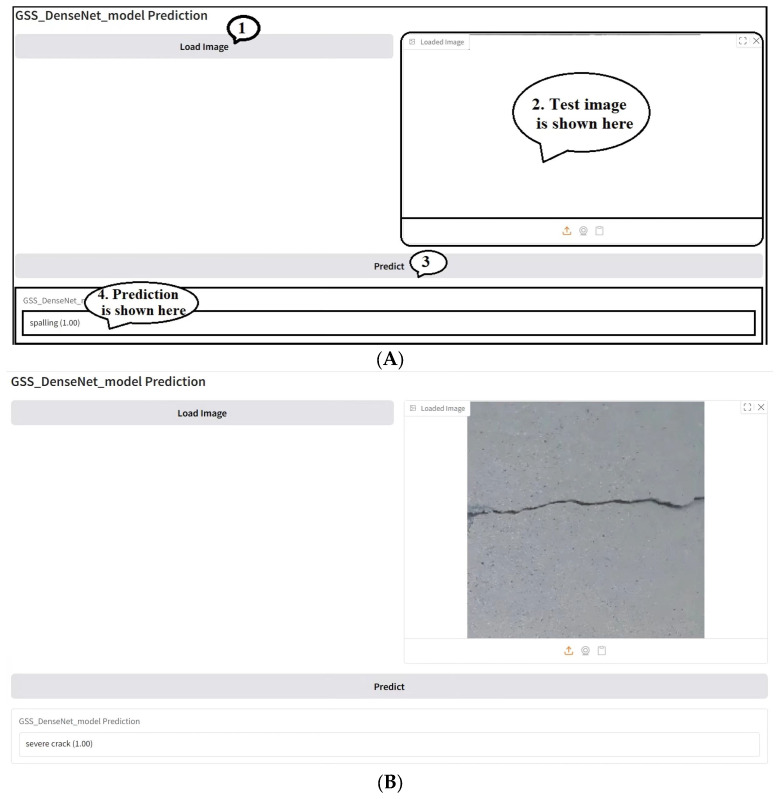

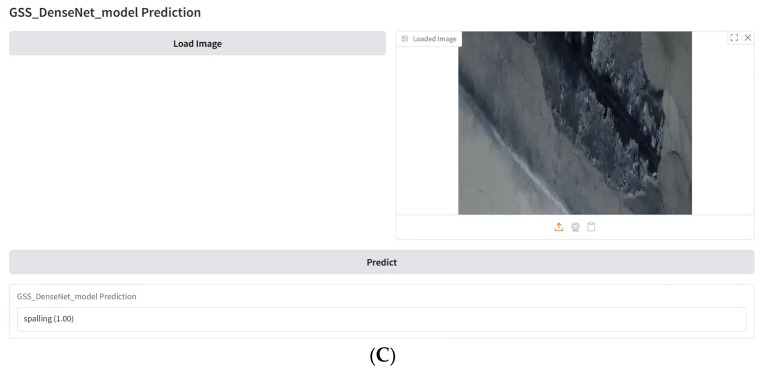
A simple Gradio-based GUI for crack type identification: (**A**) GUI design, (**B**,**C**) test cases.

In this section, a GUI that simulates real-world crack identification application is proposed. The main backend model of the designed GUI is the proposed GSSFSEDenseNet201 model. At the same time, the frontend part is a simple GUI designed using the Gradio COLAB library, a Python open-source package for developing GUIs. Figure 9 shows the GUI design and an example of a test simulation. The GUI is simple and allows users to test crack images to identify the possible crack type and confidence level, as shown in Figure 9. The designed GUI enables real-time visualization, recording, and quantitative assessment of detected defects, which can be directly utilized in measurement and instrumentation practice.

### 4.4. Model Interpretability

**Figure 10:**
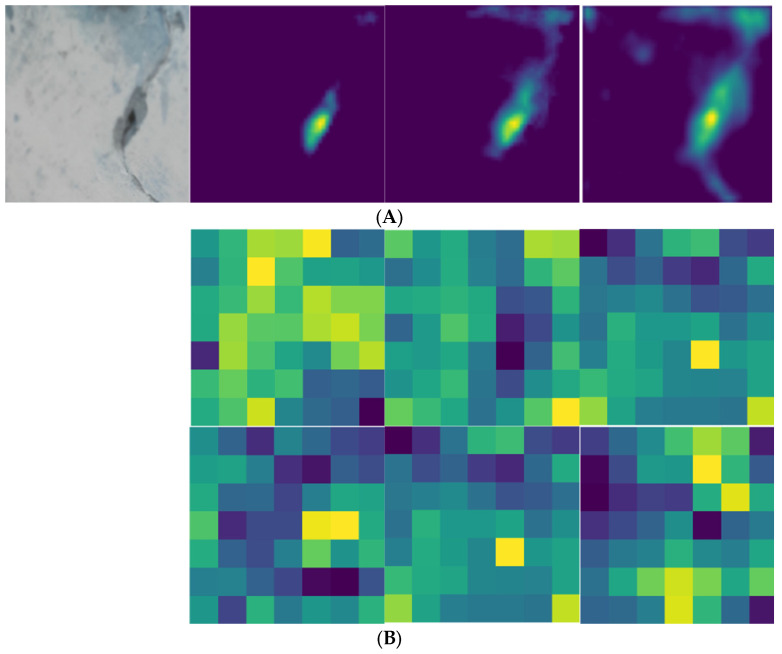
Activation maps of the proposed GSSFSEDenseNet201 model and original DenseNet201 model: (**A**) GSSFSEDenseNet201 fused maps, (**B**) DenseNet201 concatenation maps.

For better model interpretation, Figure 10 illustrates the efficiency of the proposed GSSFSEDenseNet201 model in representing the essential parts of the image, as shown in the fused maps, compared to the final concatenated map of the original DenseNet201 model. Additionally, the GSS fusion and SE blocks enable the model to effectively capture the essential features of the image. Moreover, the defective parts are well-represented in the fusion map of the proposed model; in contrast, the DenseNet201 model misses detailed features, as illustrated in Figure 10B, where the activation maps lack specific information related to the crack region within the concrete image.

### 4.5. Comparison with State-of-the-Art Methodologies and Datasets

The well-known image classification models, including VGG16, MobileNetV3, Inception-ResNet, InceptionV3, and Vision Transformer (ViT), are trained using the same dataset and training options. The results of this scenario are shown in Table 7. However, other models, including EfficientNetB0, ResNet50, and EfficientNetB3, are also trained but show bad performance. Although ViT is considered one of the most potent models, its performance is highly dependent on large-scale training data. It fails to outperform DenseNet201 or the proposed model. The ViT model splits images into non-overlapping patches, which may affect the local texture correlation of crack regions, particularly for simple and severe crack types where the crack occupies a small area of the image. Moreover, the small dataset size and variety of the proposed five crack and defect categories can limit the performance of the ViT model compared to DenseNet and the proposed architectures, which focus on small and local features. The ConvNeXt model is also trained, but it provides poor performance. However, the DenseNet core model is the best, demonstrating that our suitable selection of this model is indeed the base model for our proposed framework.

Table 8 presents a comparison between previous work and the current study, allowing for a comprehensive evaluation of the proposed methodology. Previous attempts focused on the binary classification of concrete cracks. Few studies addressed the multi-class issue [18,21]. However, in [18], they only considered two types of cracks (simple and multiple cracks), whereas in [21], the authors considered bridge crack types but implemented a computationally intensive model (modified ViT model). In our study, we considered five different types of concrete cracks using a modified DenseNet201 architecture without further computations. Our collected dataset contains various challenges, such as lighting conditions, different crack stages, and numerous concrete structures (walls, roofs, floors, columns, etc.). Moreover, we developed a novel architecture that outperformed the original DenseNet201 model and other well-known CNN-based and ViT-based models.

To further discuss the contribution of the collected dataset, Table 9 includes a detailed summary of the current proposed dataset and previous related data sources. Table 9 shows that the proposed dataset fill the gaps of previous ones in terms of three main factors: first, the dataset is the first one that contains these five different crack and severity categories which neither GYU-DET nor Del Savio provide (Ref. [3]’s dataset does not include simple or severe crack; moreover, it is designed for another mission which is object detection). Although large datasets provided in Table 8 (i.e., SDNET2018) are very common in deep learning studies, they are limited to the binary classification problem, which does not fully fit real-world applications.

**Figure 11:**
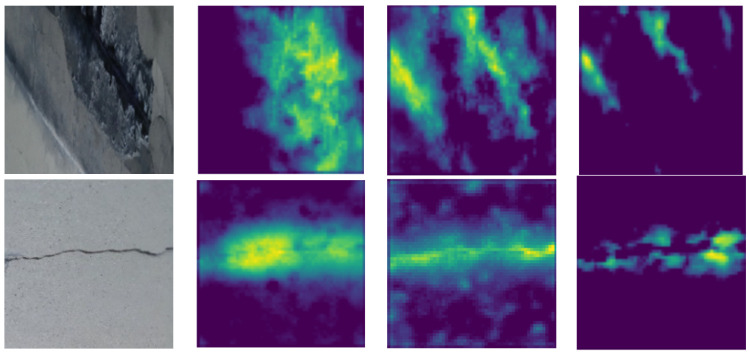

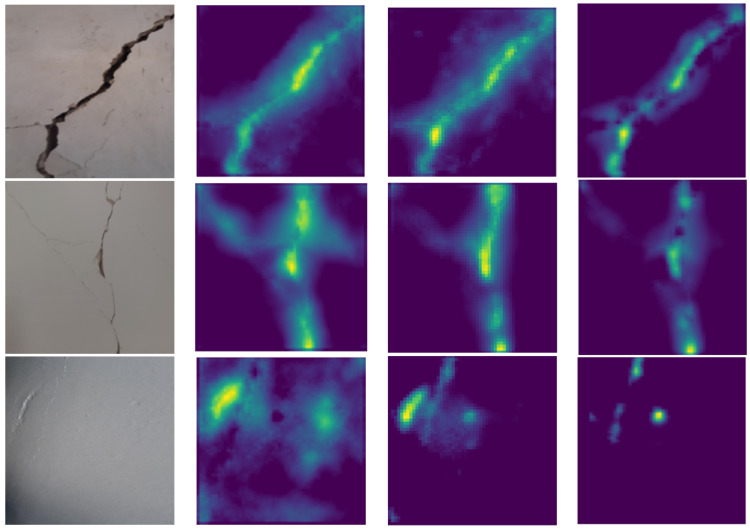
Activation maps of the proposed GSSFSEDenseNet201 using many defect-type images.

Figure 11 presents examples of different defect types and three of their corresponding fusion maps, as predicted by the GSSFSEDenseNet201 model. A visual comparison between different crack types and corresponding fusion maps proves that the proposed model effectively extracts features of the defect regions.

### 4.6. Generalization Study

**Figure 12:**
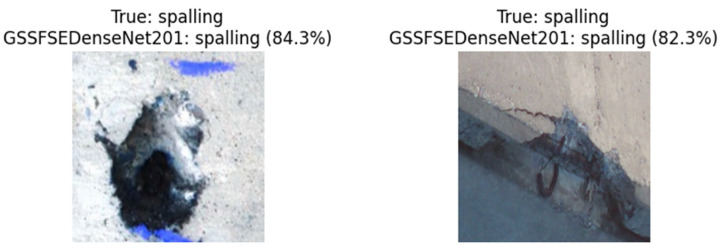

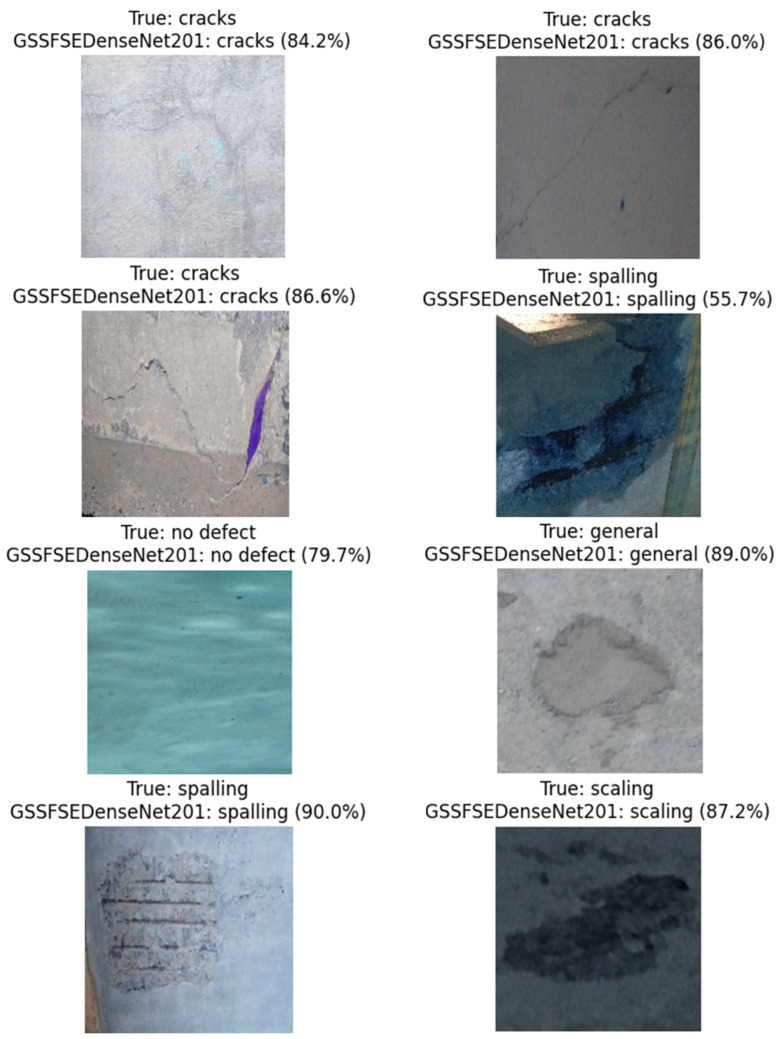
Examples of evaluating the GSSFSEDenseNet201 using the “Multi-classifier for RC bridge defects” dataset.

We examined another multi-class concrete crack dataset (“Multi-classifier for RC bridge defects” [37]) that contains six classes (“cracks”, “efflorescence”, “general”, “no defect”, “Scaling”, and “Spalling”). The validation process includes images of various categories under different imaging conditions (lighting, camera angle, and resolutions). As a result, an accuracy of 89.03% is obtained. Figure 12 presents various evaluation examples of concrete images along with their corresponding recognized categories. Again, the GSSFSEDenseNet201 demonstrates its ability to accurately identify multiple types of concrete cracks and defects.

## 5. Conclusions

This research has addressed the limited data sources for multi-class classification and the limitations of the traditional DL models in addressing changes in crack types and their distribution along concrete structures. To address the first challenge, the current study presents a novel image-based non-destructive measurement dataset of concrete defects, encompassing four distinct defect types (simple crack, severe crack, scaling, and spalling) alongside the normal case (intact). To overcome the second challenge, a novel architecture called the GSSFSEDenseNet201 model has been proposed, using the DenseNet201 base model with a Guided Semantic–Spatial Fusion module and a squeeze-and-excitation unit. While the former enhances the multi-scale feature extraction of the base model, the latter enables the model to focus on the essential defect regions within the scene (attention) and ignore other non-defect regions. Training scenarios have included training the original DenseNet201 model, the new GSSFSEDenseNet201 model, the DenseNet201 model with an added dual attention module, an ablation study by modifying the base model, and a comparison with the state-of-the-art CNN-based models. Results have proven that the proposed GSSFSEDenseNet201 model outperformed all other models in terms of all metrics. The best accuracy obtained was 95.32%, representing an improvement of 5.91% compared to the original DenseNet201 model. A GUI of the concrete defect detection method has also been designed, integrating the proposed GSSFSEDenseNet201 as a backend model. The model’s interpretability was also investigated by visualizing the fused maps of the proposed model and comparing them with the original maps of the DenseNet201 model. Comparison proved that the proposed GSSFSEDenseNet201 model effectively identified the defect regions, regardless of the defect type, whereas the original DenseNet201 model’s maps contained less-efficient defect features.

Compared to conventional field inspections accomplished by experts, the proposed GSSFSEDenseNet201 concrete crack and defect-identification methodology offers several practical advantages. Manual inspections are usually time-consuming, subjective, and require physical access to structural elements. In contrast, our non-invasive image-based GSSFSEDenseNet201 model enables accurate and precise assessment from simple images collected using mobile cameras. This reduces inspection time and improves safety. The GSSFSEDenseNet201 model also provides consistent and objective predictions, overcoming disagreements between inspectors.

Although the current study addressed the limitations in the current state-of-the-art crack detection methodologies, it still has some limitations, such as the difficulty in obtaining more defect instances and the need for model parameter reduction. Moreover, the small size of the collected dataset is a significant limitation, particularly given the five different categories of concrete cracks and defects. Future studies can address the dataset size issue and reduce the number of trainable parameters by proposing a lightweight architecture based on the same proposed methodology. Multi-modal sensing strategies can also be utilized for better concrete crack collection tasks, including depth information, thermal imaging, LiDAR, or acoustic data. Finally, real-world deployment challenges, such as domain shifts, varying imaging conditions, noise occurrence (e.g., dust or paint), and other operational constraints, should be addressed later to ensure reliability and seamless integration in real-world applications.

## Figures and Tables

**Table 1:** Performance criteria.

Metric	Definition	Equation	
**True Positives TP**	Number of correctly accepted crack-type detections (NtCd) out of all test samples (N)	$TP=\frac{N_{ctCd}}{N}$	(15)
**False Positives FP**	Number of incorrectly accepted crack-type detections (NiaCd) out of all test samples (N)	$FP=\frac{N_{iaCd}}{N}\;$	(16)
**True Negatives TN**	Number of correctly rejected non-crack-type detections (NcNd) out of all test samples (N)	$TP=\frac{N_{ctNd}}{N}$	(17)
**False Negatives FN**	Number of incorrectly rejected crack-type detections (NirCd) out of all samples (N)	$FN=\frac{N_{irCd}}{N}$	(18)
**Accuracy (ACC)**	Number of all true detections divided by the number of all test samples (N)	$ACC=\frac{TP+TN}{TP+TN+FP+FN}$	(19)
**Precision (P)**	Measure the ratio of true positives to the number of all positive samples	$P=\frac{TP}{TP+FP}$	(20)
**Recall (R)**	Measure of the ability of the trained model to correctly classify all actual positives (correctly classified crack-type samples that are actually cracks)	$R=\frac{TP}{TP+FN}$	(21)
**F1-score (F1)**	A mixture metric of precision and recall that unifies them and gives an overall performance value.	$F1=\frac{2*P*R}{P+R}$	(22)

**Table 2:** Training parameters.

Parameter	Value
Input size	224 × 224 × 3
Learning rate	1 × 10^−3^
Optimizer	Adam
Loss function	Categorical cross entropy
Training epochs	50
Early stop condition	Patience = 15
Batch size	128

**Table 3:** Performance metrics of the trained GSSFSEDenseNet201 model.

	Precision	Recall	F1 Score	Accuracy
**N**	0.9889	1.000	0.9944	0.9532
**SC**	0.9286	0.9512	0.9398	
**SV**	0.9333	0.8750	0.9032	
**SC**	0.9765	0.9765	0.9765	
**SP**	0.9310	0.9419	0.9364	
**M.avg**	0.9517	0.9489	0.9501	
**W.avg**	0.9531	0.9532	0.9530	

N: normal, SC: simple crack, SV: severe crack, SC: scaling, SP: spalling, M.avg: micro average, W.avg: weighted average.

**Table 4:** A comparison between the proposed GSSFSEDenseNet201 model and the original DenseNet201 model.

	Precision		Recall		F1 Score		Accuracy	
	New	Original	New	Original	New	Original	New	Original
**N**	0.9889	0.9545	1.000	0.9438	0.9944	0.9492	0.9532	0.8966
**SC**	0.9286	0.9079	0.9512	0.8415	0.9398	0.8734		
**SV**	0.9333	0.7746	0.8750	0.8594	0.9032	0.8148		
**SM**	0.9765	0.8736	0.9765	0.8941	0.9765	0.8837		
**SP**	0.9310	0.9524	0.9419	0.9302	0.9364	0.9412		
**M.avg**	0.9517	0.8926	0.9489	0.8938	0.9501	0.8925		
**W.avg**	0.9531	0.8994	0.9532	0.8966	0.9530	0.8973		

**Table 5:** Comparison between the proposed GSSFSEDenseNet201 model and the other models within the ablation study.

Model	Precision	Recall	F1 Score	Accuracy	AUC
**GSSFSEDenseNet201**	0.9517	0.9489	0.9501	0.9532	0.9928
**GSSFDenseNet201**	0.9209	0.9151	0.9169	0.9212	0.9877
**DenseNet201 with dual attention**	0.8970	0.8999	0.8962	0.8990	0.9843
**DenseNet201**	0.8926	0.8938	0.8973	0.8966	0.9879

**Table 6:** Effect of changing learning rate on GSSFDenseNet201’s performance.

Model	Precision	Recall	F1 Score	Accuracy	AUC
**LR = 0.001**	0.9517	0.9489	0.9501	0.9532	0.9928
**LR = 0.0001**	0.9235	0.9246	0.9218	0.9261	0.9932
**LR = 0.00001**	0.9020	0.9035	0.9022	0.9064	0.9905

**Table 7:** Evaluation of the current state-of-the-art classification models using the same dataset.

Model	Precision	Recall	F1 Score	Accuracy	AUC
**DenseNet201**	0.8926	0.8938	0.8973	0.8966	0.9879
**VGG16**	0.8329	0.8315	0.8314	0.8374	0.9687
**MobileNetV3**	0.8910	0.8919	0.8908	0.8966	0.9828
**Inception_ResNet**	0.8039	0.8018	0.8012	0.8079	0.9661
**InceptionV3**	0.8522	0.8438	0.8457	0.8522	0.9694
**ViT**	0.7762	0.7676	0.7679	0.7783	0.9520
**Efficientnetv2B0**	0.1388	0.2482	0.1622	0.2685	0.5350
** ConvNeXt **	0.1503	0.3474	0.2097	0.3744	0.5890

**Table 8:** Comparison with previous studies.

Study	Dataset	Concrete Crack Types	Methodology	Results	Limitations
**Hou et al. [15]**	DDAP: 2500 pavement distress images DDCB: 906 concrete bridge images	Crack/Non-crack	MobileNet and MobileNet-SSD	Accuracy: 97.8%	Small Dataset Binary classification
**Ritzy et al. [14]**	10,000 training images of crack and non-crack types	Crack/Non-crack	Modified InceptionV3	Binary Accuracy: 99.67%	Binary classification
**Sun et al. [16]**	2828 images collected from public sites	Crack/Non-crack	Fourier enhancement+ CNN	Accuracy: 91.6%	Binary classification
**Mayya et al. [17]**	SDNET2018: 13,620 bridge deck images	Crack/Non-crack	CNN-based fusion Transfer learning-based fusion	Accuracy: 98.62%	Binary classification
**Mayya et al. [18]**	12,000 images	Normal, Simple crack, Multiple-crack	YOLOV10 + ViT	F1-score: 90.34%	The two-stage model required more computational time
**Zhao et al. [20]**	Collected dataset	Crack/Non-crack	Revised ViT	Accuracy: 99.03%	Binary classification
**Yang et al. [21]**	2098 annotated bridge images	Corrosion, Spalling, Crack, and Rebar	MaxVit, GCN	Accuracy: 98.29%	Limited data size More complex architecture
**ALKannad et al. [22]**	SDNET2018 and METU	Crack/Non-crack	CrackVisionX	Accuracy > 99% for all scenarios	Binary classification
**by Lin et al. [23]**	SDNET2018	Crack/Non-crack	Ridgelet model, AHEO	Accuracy: 99.66%	Binary classification
**Qin et al. [24]**	SDNET2018	Crack/Non-crack	Deep belief network with IGMM	Accuracy: 90.189%	Binary classification
**Proposed GSSFSEDenseNet201 model**	Collected a dataset of 2029 images	Scaling Spalling Simple crack Severe crack Normal	Guided semantic–spatial fusion module with squeeze-and-excitation DenseNet	Accuracy: 95.32%	Limited data size

**Table 9:** Comparison with previous concrete crack datasets.

Dataset	Dataset Size	Structure	Classes	Image Variation Considered?	Binary or Multi-Class
**DDCB [15]**	906 images	Concrete bridge	Crack No-Crack	Not mentioned	Binary classification
**Ritzy et al. dataset [14]**	10,000 training images	Crack/Non-crack	Crack No-Crack	Not mentioned	Binary classification
**Sun et al. dataset [16]**	2828 images	Concrete	Crack No-Crack	Not mentioned	Binary classification
**SDNET2018 [36]**	56,000 bridge deck images	Bridge decks, walls, and pavement	Crack No-Crack	Yes	Binary classification
**Multi-classifier for RC bridge defects [25]**	1959 images	Concrete bridge	Crack, General, Normal, Efflorescence, Scaling, Spalling	Yes	Multi-class classification
**Yang et al. [21]**	2098	Bridge	Corrosion, Spalling, Crack, and Rebar	Yes	Multi-class classification
**Kumar and Ghosh dataset [30]**	3200 images	Concrete	Crack No-Crack	Yes	Binary classification
**Del Savio et al. dataset [31]**	1132 images	Beam and column structures	Crack No-Crack	Not mentioned	Binary classification
**Li dataset [3]**	11,123 images	Beam crack	Cracks, Spalling, Seepage, Honeycomb Surface, Exposed Rebar, and Holes	Yes	Object detection (YOLO annotations)
**Proposed GSSFSEDenseNet201 model**	2029 images	Various concrete structures (walls, beams, columns, floor, roofs, etc.)	Scaling Spalling Simple crack Severe crack Normal	Yes	Multi-class classification

## Data Availability

The dataset presented in this study can be tracked from the link: https://github.com/AliMayya/Multi-class-concrete-crack-dataset (accessed on 11 December 2025). Further inquiries can be directed to the corresponding author.
